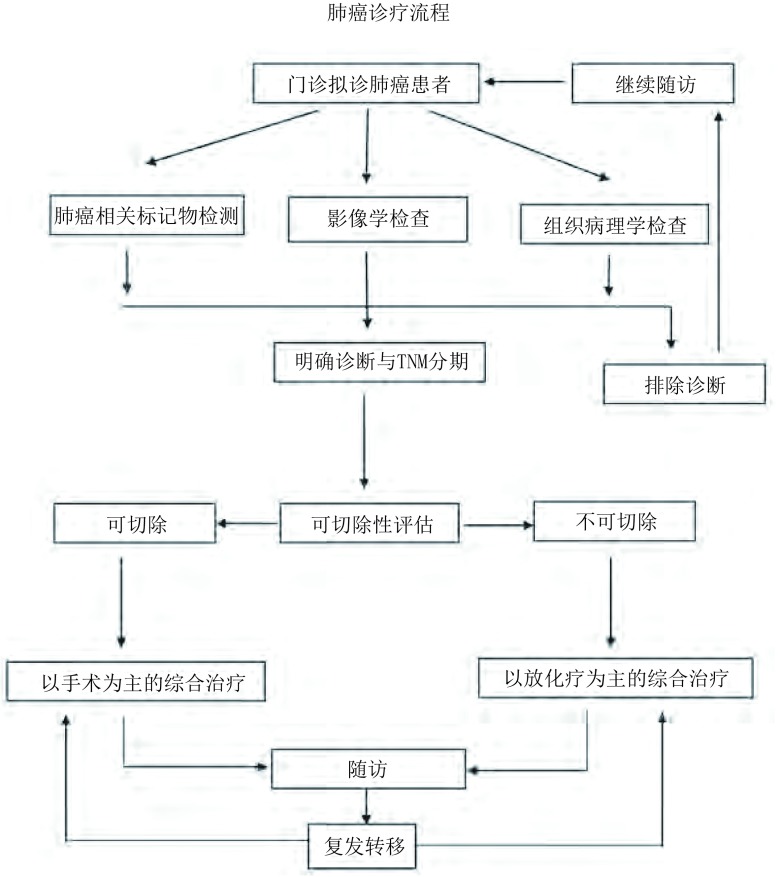# 原发性肺癌诊疗规范(2011年版)

**DOI:** 10.3779/j.issn.1009-3419.2012.12.01

**Published:** 2012-12-20

**Authors:** 修益 支, 一龙 吴, 胜林 马, 天佑 王, 长利 王, 洁 王, 远凯 石, 铀 卢, 伦旭 刘, 德若 刘, 东红 陈, 跃 杨, 祥 杜, 宏 步, 清华 周, 格宁 姜, 宝惠 韩, 刚 程, 颖 程, 顺昌 焦

**Affiliations:** 1 100053 北京，首都医科大学肺癌诊疗中心，首都医科大学宣武医院胸外科 Beijing Lung Cancer Center, Department of Thoracic Surgery, Beijing Xuanwu Hospital, Capital Medical University, Beijing 100053, China; 2 510030 广州，广东省肺癌研究所，广东省人民医院胸外科 Guangdong Lung Cancer Institute, Department of Thoracic Surgery, Guangdong General Hospital, Guangzhou 510030, China; 3 310022 杭州，浙江省肿瘤医院放疗科 Department of Radiation Therapy, Zhejiang Cancer Hospital, Hangzhou 310022, China; 4 100050 北京，北京友谊医院胸外科 Department of Thoracic Surgery, Beijing Friendship Hospital, Beijing 100050, China; 5 300060 天津，天津市肿瘤医院肺部肿瘤科 Department of Lung Cancer Surgery, Tianjin Cancer Hospital, Tianjin 300060, China; 6 100142 北京，北京大学肿瘤医院胸部肿瘤内科 Department of Thoracic Medical Oncology, Beijing Cancer Hospital, Peking University, Beijing 100142, China; 7 100021 北京，中国医学科学院肿瘤医院肿瘤内科 Department of Medical Oncology, Cancer Hospital, Chinese Aacademy of Medical Sciences and Peking Union Medical College, Beijing 100021, China; 8 610041 成都，四川大学华西医院肿瘤科 Department of Medical Oncology, West China Hospital, Sichuan University, Chengdu 610041, China; 9 610041 成都，四川大学华西医院胸外科 Department of Thoracic Surgery, West China Hospital, Sichuan University, Chengdu 610041, China; 10 100029 北京，卫生部中日友好医院胸外科 Department of Thoracic Surgery, China-Japan Friendship Hospital, Beijing 100029, China; 11 100142 北京，北京大学肿瘤医院胸外科 Department of Thoracic Surgery, Beijing Cancer Hospital, Peking University, Beijing 100142, China; 12 200032 上海，复旦大学肿瘤医院病理科 Department of Pathology, Cancer Hospital, Fudan University, Shanghai 200032, China; 13 610041 成都，四川大学华西医院病理科 Department of Pathology, West China Hospital, Sichuan University, Chengdu 610041, China; 14 300052 天津，天津医科大学总医院肺外科 Department of Thoracic Surgery, Tianjin Medical University General Hospital, Tianjin 300052, China; 15 200433 上海，同济大学肺科医院胸外科 Department of Thoracic Surgery, Shanghai Pulmonary Hospital, Tongji University, Shanghai 200433, China; 16 200030 上海，上海交通大学附属胸科医院呼吸内科 Department of Respiratory Medicine, Shanghai Chest Hospital, Shanghai Jiao Tong University, Shanghai 200030, China; 17 100005 北京，卫生部北京医院肿瘤内科 Department of Medical Oncology, Beijing Hospital, Beijing 100005, China; 18 130012 长春，吉林省肿瘤医院肿瘤内科 Department of Medical Oncology, Jilin Cancer Hospital, Changchun 130012, China; 19 100853 北京，解放军总医院肿瘤内科 Department of Medical Oncology, General Hospital of Chinese PLA, Beijing 100853, China

## 一 概述

原发性肺癌(以下简称肺癌)是我国最常见的恶性肿瘤之一。2010年卫生统计年鉴显示，2005年，肺癌死亡率占我国恶性肿瘤死亡率的第1位。

为进一步规范我国肺癌诊疗行为，提高医疗机构肺癌诊疗水平，改善肺癌患者预后，保障医疗质量和医疗安全，特制定本规范。

## 二 诊断技术与应用

### 一 高危因素

有吸烟史并且吸烟指数大于400支/年、高危职业接触史(如接触石棉)以及肺癌家族史等，年龄在45岁以上者，是肺癌的高危人群。

### 二 临床表现

1.肺癌早期可无明显症状。当病情发展到一定程度时，常出现以下症状：

(1) 刺激性干咳。

(2) 痰中带血或血痰。

(3) 胸痛。

(4) 发热。

(5) 气促。

当呼吸道症状超过两周，经治疗不能缓解，尤其是痰中带血、刺激性干咳，或原有的呼吸道症状加重时，要高度警惕肺癌存在的可能性。

2.当肺癌侵及周围组织或转移时，可出现如下症状：

(1) 癌肿侵犯喉返神经出现声音嘶哑。

(2) 癌肿侵犯上腔静脉，出现面、颈部水肿等上腔静脉梗阻综合征表现。

(3) 癌肿侵犯胸膜引起胸膜腔积液，往往为血性；大量积液可以引起气促。

(4) 癌肿侵犯胸膜及胸壁，可以引起持续剧烈的胸痛。

(5) 上叶尖部肺癌可侵入和压迫位于胸廓入口的器官组织，如第一肋骨、锁骨下动、静脉、臂丛神经、颈交感神经等，产生剧烈胸痛，上肢静脉怒张、水肿、臂痛和上肢运动障碍，同侧上眼脸下垂、瞳孔缩小、眼球内陷、面部无汗等颈交感神经综合征表现。

(6) 近期出现的头痛、恶心、眩晕或视物不清等神经系统症状和体征应当考虑脑转移的可能。

(7) 持续固定部位的骨痛、血浆碱性磷酸酶或血钙升高应当考虑骨转移的可能。

(8) 右上腹痛、肝肿大、碱性磷酸酶、谷草转氨酶、乳酸脱氢酶或胆红素升高应当考虑肝转移的可能。

(9) 皮下转移时可在皮下触及结节。

(10)血行转移到其它器官可出现转移器官的相应症状。

### 三 体格检查

1.多数肺癌患者无明显相关阳性体征。

2.患者出现原因不明，久治不愈的肺外征象，如杵状指(趾)、非游走性肺性关节疼痛、男性乳腺增生、皮肤黝黑或皮肌炎、共济失调、静脉炎等。

3.临床表现高度可疑肺癌的患者，体检发现声带麻痹、上腔静脉梗阻综合征、Horner征、Pancoast综合征等提示局部侵犯及转移的可能。

4.临床表现高度可疑肺癌的患者，体检发现肝肿大伴有结节、皮下结节、锁骨上窝淋巴结肿大等提示远处转移的可能。

### 四 影像检查

1.胸部X线检查：胸片是早期发现肺癌的一个重要手段，也是术后随访的方法之一。

2.胸部CT检查：胸部CT可以进一步验证病变所在的部位和累及范围，也可大致区分其良、恶性，是目前诊断肺癌的重要手段。低剂量螺旋胸部CT可以有效地发现早期肺癌，而CT引导下经胸肺肿物穿刺活检是重要的获取细胞学、组织学诊断的技术。

3.B型超声检查：主要用于发现腹部重要器官以及腹腔、腹膜后淋巴结有无转移，也用于双锁骨上窝淋巴结的检查；对于邻近胸壁的肺内病变或胸壁病变，可鉴别其囊、实性及进行超声引导下穿刺活检；超声还常用于胸水抽取定位。

4.MRI检查：MRI检查对肺癌的临床分期有一定价值，特别适用于判断脊柱、肋骨以及颅脑有无转移。

5.骨扫描检查：用于判断肺癌骨转移的常规检查。当骨扫描检查提示骨可疑转移时，可对可疑部位进行MRI检查验证。

6.PET-CT检查：不推荐常规使用。在诊断肺癌纵隔淋巴结转移时较CT的敏感性、特异性高。

### 五 内窥镜检查

1.纤维支气管镜检查：纤维支气管镜检查技术是诊断肺癌最常用的方法，包括纤支镜直视下刷检、活检以及支气管灌洗获取细胞学和组织学诊断。上述几种方法联合应用可以提高检出率。

2.经纤维支气管镜引导透壁穿刺纵隔淋巴结活检术(transbronchial needle aspiration, TBNA)和纤维超声支气管镜引导透壁淋巴结穿刺活检术(EBUS-TBNA)：TBNA有助于治疗前肺癌TNM分期的精确N_2_分期。但不作为常规推荐的检查方法，有条件的医院应当积极开展。EBUS-TBNA更能就肺癌N_1_和N_2_的精确病理诊断提供安全可靠的支持。

3.纵隔镜检查：作为确诊肺癌和评估N分期的有效方法，是目前临床评价肺癌纵隔淋巴结状态的金标准。尽管CT、MRI以及近年应用于临床的PET-CT能够对肺癌治疗前的N分期提供极有价值的证据，但仍然不能取代纵隔镜的诊断价值。

4.胸腔镜检查：胸腔镜可以准确地进行肺癌诊断和分期，对于经纤维支气管镜和经胸壁肺肿物穿刺针吸活检术(transthoracic needle aspiration, TTNA)等检查方法无法取得病理标本的早期肺癌，尤其是肺部微小结节病变行胸腔镜下病灶切除，即可以明确诊断。对于中晚期肺癌，胸腔镜下可以行淋巴结、胸膜和心包的活检，胸水及心包积液的细胞学检查，为制定全面治疗方案提供可靠依据。

### 六 其它检查技术

1.痰细胞学检查：痰细胞学检查是目前诊断肺癌简单方便的无创伤性诊断方法之一，连续三天留取清晨深咳后的痰液进行痰细胞学涂片检查可以获得细胞学的诊断。

2.经胸壁肺内肿物穿刺针吸活检术：TTNA可以在CT或B超引导下进行，在诊断周围型肺癌的敏感度和特异性上均较高。

3.胸腔穿刺术：当胸水原因不清时，可以进行胸腔穿刺，以进一步获得细胞学诊断，并可以明确肺癌的分期。

4.胸膜活检术：当胸水穿刺未发现细胞学阳性结果时，胸膜活检可以提高阳性检出率。

5.浅表淋巴结活检术：对于肺部占位病变或已明确诊断为肺癌的患者，如果伴有浅表淋巴结肿大，应当常规进行浅表淋巴结活检，以获得病理学诊断，进一步判断肺癌的分期，指导临床治疗。

### 七 血液免疫生化检查

1.血液生化检查：对于原发性肺癌，目前无特异性血液生化检查。肺癌患者血浆碱性磷酸酶或血钙升高考虑骨转移的可能，血浆碱性磷酸酶、谷草转氨酶、乳酸脱氢酶或胆红素升高考虑肝转移的可能。

2.血液肿瘤标志物检查：目前尚无特异性肺癌标志物应用于临床诊断，故不作为常规检查项目，但有条件的医院可以酌情进行如下检查，作为肺癌评估的参考：

(1) 癌胚抗原(carcinoembryonic antigen, CEA)：目前血清中CEA的检查主要用于判断肺癌预后以及对治疗过程的监测。

(2) 神经特异性烯醇化酶(neurone specific enolase, NSE)：是小细胞肺癌首选标志物，用于小细胞肺癌的诊断和治疗反应监测。

(3) 细胞角蛋白片段19(cytokeratin fragment, CYFRA21-1)：对肺鳞癌诊断的敏感性、特异性有一定参考意义。

(4) 鳞状细胞癌抗原(squarmous cell carcinoma antigen, SCC)：对肺鳞状细胞癌疗效监测和预后判断有一定价值。

### 八 组织学诊断

组织病理学诊断是肺癌确诊和治疗的依据。活检确诊为肺癌时，应当进行规范化治疗。如因活检取材的限制，活检病理不能确定病理诊断时，建议临床医师重复活检或结合影像学检查情况进一步选择诊疗方案，必要时临床与病理科医师联合会诊确认病理诊断。

### 九 肺癌的鉴别诊断

1.良性肿瘤：常见的有肺错构瘤、支气管肺囊肿、巨大淋巴结增生、炎性肌母细胞瘤、硬化性血管瘤、结核瘤、动静脉瘘和肺隔离症等。这些良性病变在影像检查上各有其特点，若与恶性肿瘤不易区别时，应当考虑手术切除。

2.结核性病变：是肺部疾病中较常见也是最容易与肺癌相混淆的病变。临床上容易误诊误治或延误治疗。对于临床上难于鉴别的病变，应当反复做痰细胞学检查、纤维支气管镜检查及其它辅助检查，直至开胸探查。在明确病理或细胞学诊断前禁忌行放射治疗(以下简称放疗)或化学药物治疗(以下简称化疗)，但可进行诊断性抗结核治疗及密切随访。结核菌素试验阳性不能作为排除肺癌的指标。

3.肺炎：大约有1/4的肺癌早期以肺炎的形式出现。对起病缓慢，症状轻微，抗炎治疗效果不佳或反复发生在同一部位的肺炎应当高度警惕有肺癌可能。

4.其它：包括发生在肺部的一些少见、罕见的良、恶性肿瘤，如肺纤维瘤、肺脂肪瘤等，术前往往难以鉴别。

## 三 病理评估

### 一 肺癌的标本固定标准

1.固定液：推荐使用10%中性福尔马林固定液，避免使用含有重金属的固定液。

2.固定液量：必须为所固定标本体积的10倍或以上。

3.固定温度：常温。

4.按照收到标本时肿瘤的部位和状态，可有两种选择：

(1) 标本直接放入10%中性福尔马林固定。

(2) 必要时从支气管注入足够量的10%中性福尔马林固定液，结扎或钳住支气管，固定过夜。

5.固定时间：活检标本：≥6小时，≤48小时；手术标本：≥12小时，≤48小时。

### 二 取材要求

1.活检标本。

(1) 核对临床送检活检标本数量，送检活检标本必须全部取材。

(2) 每个蜡块内包括不超过5粒活检标本。

(3) 将标本包于纱布或柔软的透水纸中以免丢失。

2.手术标本。

(1) 局部肺脏切除标本(肺段切除和肺楔形切除标本)。

① 去除外科缝合线或金属钉。

② 记录标本的大小以及胸膜表面的情况。

③ 垂直切缘切取肺实质组织块，描述肿块的大小、切面情况(伴有/无出血/坏死/空洞形成)及其与胸膜和肺实质的关系以及肿块边缘与切缘的距离。

④ 根据病变的部位和大小取1-4块组织，切取肿瘤与胸膜、肿瘤与肺实质切缘的组织块。

⑤ 切取非肿瘤部位肺组织。

(2) 肺叶及全肺切除标本。

① 检查肺的五大基本结构：气道、肺实质、胸膜、血管和淋巴结。测量大小，以肺门给标本定位。

② 取支气管切缘、血管切缘及胸膜缘。

③ 全肺切除标本，查找肺门淋巴结。

④ 按照收到标本时肿瘤的部位和状态，可有两种选择：一是用剪刀沿纵轴打开所有的主支气管及其分支，以能最好地暴露病变与周围肺组织结构关系的平面剖开肺组织。二是对主支气管内注入福尔马林的标本，每隔0.5 cm-1.0 cm切开，切面应为额平面，垂直于肺门。

⑤ 描述肿瘤大小、切面情况(伴有/无出血/坏死/空洞形成)、在肺叶和肺段内的位置以及与支气管的关系、病变范围(局灶或转移)以及切除是否充分。视肿瘤大小、发生部位、范围等充分取材(常规4块)，并切取能够显示肿瘤与周围肺组织关系的组织(常规2块)。

⑥ 切取非肿瘤部位肺组织。

(3) 淋巴结。

建议外科医师采用美国癌症联合会(American Joint Committee on Cancer, AJCC)关于术中分期系统的区域淋巴结分组方式(N)对淋巴结进行分组。N_2_淋巴结通常单独送检并由外科医师进行准确的分组，因此应当单独报告这些淋巴结。肺切除标本常附带的N_2_淋巴结，应当根据具体部位区分。沿支气管查找肺门软组织及肺实质中的淋巴结，查找到的全部淋巴结均需取材，记录部位。所有肉眼阴性的淋巴结应当完整送检，肉眼阳性的淋巴结可部分切取送检。

(4) 推荐取材组织块体积：不大于2 cm×1.5 cm×0.3 cm。

3.取材后标本处理原则和保留时限。

(1) 剩余标本的保存。取材剩余组织保存在标准固定液中，并始终保持充分的固定液量和甲醛浓度，避免标本干枯或因固定液量不足或浓度降低而致组织腐变；以备在病理诊断报告签发后接到临床反馈信息时复查大体标本或补充取材。

(2) 剩余标本处理的时限。建议在病理诊断报告签发1个月后，未接到临床反馈信息，未发生因外院会诊意见分歧而要求复审等情形后，由医院自行处理。

4.病理类型。

(1) 肺癌的大体类型：直接描写肿瘤的部位，记录肿瘤距隆突的长度。

(2) 肺癌的组织学类型：参照2004版WHO肺癌组织学分类(附件1)。

5.病理报告内容。

(1) 活检标本的病理报告内容和要求。

① 患者基本信息及送检信息。

② 如有上皮内瘤变(异型增生)，报告分级。

③ 如有癌变，区分组织学类型。

(2)手术标本的病理报告内容和要求。

 ①患者基本信息及送检信息。

② 大体情况：测量肺的大小，描述其它附带的结构；描述肿瘤与肺叶、肺段和(或)主气道和胸膜的关系；描述肿瘤距支气管切缘的远近，必要时说明距其它切缘的远近(即胸壁软组织肺门血管)；描述肿瘤大小，是否有卫星结节；描述非肿瘤性肺组织。

③ 诊断报告内容：一是肿瘤部位：肿瘤位于哪一侧肺、肺叶，如果可能，说明具体的肺段。二是手术类型：即肺段切除、肺叶切除、肺切除，包括部分肺切除。三是组织学类型，具体包括以下几个方面：组织学分级、切缘的组织学评价、累及胸膜情况、血管淋巴管的侵润情况、神经周围的侵润情况、淋巴结转移情况等。

④ 鉴别诊断相关的主要免疫组化项目：鳞状细胞癌重点筛查CK14、CK5/6、34βE12和p63；肺腺癌重点筛查CK7和TTF-l；肺神经内分泌癌重点筛查CK18、AE1/AE3、CD56、CgA、NSE和Syn。

⑤ 需要时可选做用药及预后相关的检测项目：HER2、VEGF、p53、p170、Top2A、PCNA、Ki-67。

完整的病理报告的前提是临床医师填写详细的病理诊断申请单，详细描述手术所见及相关临床辅助检查结果并清楚标记淋巴结。临床医师与病理医师的相互交流、信任和配合是建立正确分期和指导临床治疗的基础。

## 四 肺癌的分期

### 一 非小细胞肺癌

目前非小细胞肺癌的TNM分期采用国际肺癌研究协会(International Association for the Study of Lung Cancer, IASLC)2009年第七版分期标准(IASLC 2009)。

1.肺癌TNM分期中T、N、M的定义。

(1) 原发肿瘤(T)。

T_X_：原发肿瘤不能评估，或痰、支气管冲洗液找到癌细胞但影像学或支气管镜没有可见的肿瘤。

T_0_：没有原发肿瘤的证据。

Tis：原位癌。

T_1_：肿瘤最大径≤3 cm，周围被肺或脏层胸膜所包绕，支气管镜下肿瘤侵犯没有超出叶支气管(即没有累及主支气管)。

T_1a_：肿瘤最大径≤2 cm。

T_1b_：肿瘤最大径 > 2 cm且≤3 cm。

T_2_：肿瘤大小或范围符合以下任何一项：肿瘤最大径 > 3 cm; 但不超过7 cm；累及主支气管，但距隆突≥2 cm；累及脏层胸膜；扩展到肺门的肺不张或阻塞性肺炎，但不累及全肺。

T_2a_：肿瘤最大径≤5 cm，且符合以下任何一点：肿瘤最大径 > 3 cm；累及主支气管，但距隆突≥2 cm；累及脏层胸膜；扩展到肺门的肺不张或阻塞性肺炎，但不累及全肺。

T_2b_：肿瘤最大径 > 5 cm且≤7 cm。

T_3_：任何大小的肿瘤已直接侵犯了下述结构之一者：胸壁(包括肺上沟瘤)、膈肌、纵隔胸膜、心包；或肿瘤位于距隆突2 cm以内的主支气管，但尚未累及隆突；或全肺的肺不张或阻塞性肺炎。肿瘤最大径 > 7 cm；与原发灶同叶的单个或多个的卫星灶。

T_4_：任何大小的肿瘤已直接侵犯了下述结构之一者：纵隔、心脏、大血管、气管、食管、喉返神经、椎体、隆突；或与原发灶不同叶的单发或多发病灶。

(2) 区域淋巴结(N)。

N_X_：区域淋巴结不能评估。

N_0_：无区域淋巴结转移。

N_1_：转移至同侧支气管旁淋巴结和(或)同侧肺门淋巴结，和肺内淋巴结，包括原发肿瘤直接侵犯。

N_2_：转移至同侧纵隔和(或)隆突下淋巴结。

N_3_：转移至对侧纵隔、对侧肺门淋巴结、同侧或对侧斜角肌或锁骨上淋巴结。

(3) 远处转移(M)。

M_X_：远处转移不能评估。

M_0_：无远处转移。

M_1_：有远处转移。

M_1a_：胸膜播散(包括恶性胸膜积液、恶性心包积液、胸膜转移结节)；对侧肺叶的转移性结节。

M_1b_：胸腔外远处转移。

大部分肺癌患者的胸腔积液(或心包积液)是由肿瘤所引起的。但如果胸腔积液(或心包积液)的多次细胞学检查未能找到癌细胞，胸腔积液(或心包积液)又是非血性或非渗出性的，临床判断该胸腔积液(或心包积液)与肿瘤无关，这种类型的胸腔积液(或心包积液)不影响分期。

2.肺癌TNM分期(IASLC 2009)。

**Table:** 肺癌TNM分期(IASLC 2009)

分期	TNM
隐形肺癌	T_x_，N_0_，M_0_
0	T_is_，N_0_，M_0_
ⅠA	T_1a, b_，N_0_，M_0_
ⅠB	T_2a_，N_0_，M_0_
ⅡA	T_1a, b_，N_1_，M_0_
	T_2a_，N_1_，M_0_
	T_2b_，N_0_，M_0_
ⅡB	T_2_，N_1_，M_0_
	T_3_，N_0_，M_0_
ⅢA	T_1_，N_2_，M_0_
	T_2_，N_2_，M_0_
	T_3_，N_1_，M_0_
	T_3_，N_2_，M_0_
	T_4_，N_0_，M_0_
	T_4_，N_1_，M_0_
ⅢB	T_4_，N_2_，M_0_
	任何T，N_3_，M_0_
Ⅳ	任何T，任何N，M_1a, b_

### 二 小细胞肺癌

小细胞肺癌分期：对于接受非手术的患者采用局限期和广泛期分期方法，对于接受外科手术的患者采用国际肺癌研究协会(IASLC)2009年第七版分期标准。

## 五 治疗

### 一 治疗原则

应当采取综合治疗的原则，即：根据患者的机体状况，肿瘤的细胞学、病理学类型，侵及范围(临床分期)和发展趋向，采取多学科综合治疗(multi-disciplinary team, MDT)模式，有计划、合理地应用手术、化疗、放疗和生物靶向等治疗手段，以期达到根治或最大程度控制肿瘤，提高治愈率，改善患者的生活质量，延长患者生存期的目的。目前肺癌的治疗仍以手术治疗、放射治疗和药物治疗为主。

### 二 外科手术治疗

1.手术治疗原则。

手术切除是肺癌的主要治疗手段，也是目前临床治愈肺癌的唯一方法。肺癌手术分为根治性手术与姑息性手术，应当力争根治性切除。以期达到最佳、彻底的切除肿瘤，减少肿瘤转移和复发，并且进行最终的病理TNM分期，指导术后综合治疗。对于可手术切除的肺癌应当遵守下列外科原则：

(1) 全面的治疗计划和必要的影像学检查(临床分期检查)均应当在非急诊手术治疗前完成。充分评估决定手术切除的可能性并制订手术方案。

(2) 尽可能做到肿瘤和区域淋巴结的完全性切除；同时尽量保留有功能的健康肺组织。

(3) 电视辅助胸腔镜外科手术(video-assisted thoracoscopic surgery, VATS)是近年来发展较快的微创手术技术，主要适用于Ⅰ期肺癌患者。

(4) 如果患者身体状况允许，应当行解剖性肺切除术(肺叶切除、支气管袖状肺叶切除或全肺切除术)。如果身体状况不允许，则行局限性切除：肺段切除(首选)或楔形切除，亦可选择VATS术式。

(5) 完全性切除手术(R_0_手术)除完整切除原发病灶外，应当常规进行肺门和纵隔各组淋巴结(N_1_和N_2_淋巴结)切除并标明位置送病理学检查。最少对3个纵隔引流区(N_2_站)的淋巴结进行取样或行淋巴结清除，尽量保证淋巴结整块切除。建议右胸清除范围为：2R、3a, 3p、4R、7-9组淋巴结以及周围软组织；左胸清除范围为：4L、5-9组淋巴结以及周围软组织。

(6) 术中依次处理肺静脉、肺动脉，最后处理支气管。

(7) 袖状肺叶切除术在术中快速病理检查保证切缘(包括支气管、肺动脉或静脉断端)阴性的情况下，尽可能保留更多肺功能(包括支气管或肺血管)，术后患者生活质量优于全肺切除术患者。

(8) 肺癌完全性切除术后6个月复发或孤立性肺转移者，在排除肺外远处转移情况下，可行复发侧余肺切除或肺转移病灶切除。

(9) 心肺功能等机体状况经评估无法接受手术的Ⅰ期和Ⅱ期的患者，可改行根治性放疗、射频消融治疗以及药物治疗等。

2.手术适应证。

(1) Ⅰ、Ⅱ期和部分ⅢA期(T_3_N_1-2_M_0_;T_1-2_N_2_M_0_；T_4_N_0-1_M_0_可完全性切除)非小细胞肺癌和部分小细胞肺癌(T_1_-2N_0-1_M_0_)。

(2) 经新辅助治疗(化疗或化疗加放疗)后有效的N_2_期非小细胞肺癌。

(3) 部分ⅢB期非小细胞肺癌(T_4_N_0-1_M_0_)如能局部完全切除肿瘤者，包括侵犯上腔静脉、其它毗邻大血管、心房、隆凸等。

(4) 部分Ⅳ期非小细胞肺癌，有单发对侧肺转移，单发脑或肾上腺转移者。

(5) 临床高度怀疑肺癌的肺内结节，经各种检查无法定性诊断，可考虑手术探查。

3.手术禁忌证

(1) 全身状况无法耐受手术，心、肺、肝、肾等重要脏器功能不能耐受手术者。

(2) 绝大部分诊断明确的Ⅳ期、大部分IIIB期和部分IIIA期非小细胞肺癌，以及分期晚于T1-2N0-1M0期的小细胞肺癌。

## 三 放射治疗

肺癌放疗包括根治性放疗、姑息放疗、辅助放疗和预防性放疗等。

1.放疗的原则。

(1) 对根治性放疗适用于KPS评分≥70分(Karnofsky评分见附件2)的患者，包括因医源性或/和个人因素不能手术的早期非小细胞肺癌、不可切除的局部晚期非小细胞肺癌以及局限期小细胞肺癌。

(2) 姑息性放疗适用于对晚期肺癌原发灶和转移灶的减症治疗。对于非小细胞肺癌单发脑转移灶手术切除患者可以进行全脑放疗。

(3) 辅助放疗适应于术前放疗、术后切缘阳性的患者，对于术后pN_2_阳性的患者，鼓励参加临床研究。

(4) 术后放疗设计应当参考患者手术病理报告和手术记录。

(5) 预防性放疗适用于全身治疗有效的小细胞肺癌患者全脑放疗。

(6) 放疗通常联合化疗治疗肺癌，因分期、治疗目的和患者一般情况的不同，联合方案可选择同步放化疗、序贯放化疗。建议同步放化疗方案为EP和含紫衫类方案。

(7) 接受放化疗的患者，潜在毒副反应会增大，治疗前应当告知患者；放疗设计和实施时，应当注意对肺、心脏、食管和脊髓的保护；治疗过程中应当尽可能避免因毒副反应处理不当导致的放疗非计划性中断。

(8) 建议采用三维适型放疗(3DCRT)与调强放疗技术(IMRT)等先进的放疗技术。

(9) 接受放疗或放化疗的患者，治疗休息期间应当予以充分的监测和支持治疗。

2.非小细胞肺癌(non-small cell lung cancer, NSCLC)放疗的适应证。

放疗可用于因身体原因不能手术治疗的早期NSCLC患者的根治性治疗、可手术患者的术前、术后辅助治疗、局部晚期病灶无法切除患者的局部治疗以及晚期不可治愈患者的重要姑息治疗方式。

Ⅰ期不能接受手术治疗的NSCLC患者，放射治疗是有效的局部控制病灶的手段之一。对于接受手术治疗的NSCLC患者，如果术后病理手术切缘阴性而纵隔淋巴结阳性(pN_2_)，除了常规接受术后辅助化疗外，也建议加用术后放疗。对于切缘阳性的pN_2_肿瘤，如果患者身体许可，建议采用术后同步放化疗。对切缘阳性的患者，放疗应当尽早开始。

对于因身体原因不能接受手术的Ⅱ期-Ⅲ期NSCLC患者，如果身体条件许可，应当给予适形放疗结合同步化疗。对有治愈希望的患者，在接受放疗或同步放化疗时，通过更为适行的放疗计划和更为积极的支持治疗，尽量减少治疗时间的中断或治疗剂量的降低。

对于有广泛转移的Ⅳ期NSCLC患者，部分患者可以接受原发灶和转移灶的放射治疗以达到姑息减症的目的。

3.小细胞肺癌(small cell lung cancer, SCLC)放疗的适应证。

局限期SCLC经全身化疗后部分患者可以达到完全缓解，但是如果不加用胸部放疗，胸内复发的风险很高，加用胸部放疗不仅可以明显降低局部复发率，而且死亡风险也明显降低。

对于广泛期SCLC患者，远处转移灶经化疗控制后加用胸部放疗也可以提高肿瘤控制率，延长生存期。

如果病情许可，小细胞肺癌的放射治疗应当尽早开始，可以考虑与化疗同步进行。如果病灶巨大，放射治疗导致肺损伤的风险过高的话，也可以考虑先采用2个-3个周期的化疗，然后尽快开始放疗。

4.预防性脑照射。

局限期小细胞肺癌患者，在胸内病灶经治疗达到完全缓解后推荐加用预防性脑照射。广泛期小细胞肺癌在化疗有效的情况下，加用预防性脑照射亦可降低小细胞肺癌脑转移发生的风险。

而非小细胞肺癌全脑预防照射的决定应当是医患双方充分讨论，根据每个患者的情况权衡利弊后确定。

5.晚期肺癌患者的姑息放疗。

晚期肺癌患者的姑息放疗主要目的是为了解决因原发灶或转移灶导致的局部压迫症状、骨转移导致的疼痛、以及脑转移导致的神经症状等。对于此类患者可以考虑采用低分割照射技术，使患者更方便地得到治疗，同时可以更迅速地缓解症状。

6.治疗效果。

放射治疗的疗效评价参照WHO实体瘤疗效评价标准(附件3)或RECIST疗效评价标准(附件4)。

7.防护。

采用常规的放疗技术，应当注意对肺、心脏、食管和脊髓的保护，以避免对身体重要器官的严重放射性损伤。急性放射性肺损伤参照RTOG分级标准(附件5)。

## 四 肺癌的药物治疗

肺癌的药物治疗包括化疗和分子靶向药物治疗[表皮生长因子受体酪氨酸激酶抑制剂(epidermal growth factor receptor-tyrosine kinase inhibitor, EGFR-TKI)治疗]。化疗分为姑息化疗、辅助化疗和新辅助化疗，应当严格掌握临床适应证，并在肿瘤内科医师的指导下施行。化疗应当充分考虑患者病期、体力状况、不良反应、生活质量及患者意愿，避免治疗过度或治疗不足。应当及时评估化疗疗效，密切监测及防治不良反应，并酌情调整药物和(或)剂量。

化疗的适应证为：PS评分≤2分(附件6，ZPS评分，5分法)，重要脏器功能可耐受化疗，对于SCLC的化疗PS评分可放宽到3分。鼓励患者参加临床试验。

1.晚期NSCLC的药物治疗。

(1) 一线药物治疗。

含铂两药方案为标准的一线治疗；表皮生长因子受体(epidermal growth factor receptor, EGFR)突变患者，可选择靶向药物的治疗；有条件者，在化疗基础上可联合抗肿瘤血管药物。目前可选用的化疗药物见附件7。对一线治疗达到疾病控制(CR+PR+SD)的患者，有条件者可选择维持治疗。

(2) 二线药物治疗。二线治疗可选择的药物包括多西紫杉醇、培美曲塞以及靶向药物EGFR-TKI。

(3) 三线药物治疗。可选择EGFR-TKI或进入临床试验。

2.不能手术切除的NSCLC的药物治疗。

推荐放疗、化疗联合，根据具体情况可选择同步或序贯放化疗。同步治疗推荐化疗药物为足叶乙甙/顺铂或卡铂(EP/EC)与紫杉醇或多西紫杉醇/铂类。序贯治疗化疗药物见一线治疗。

3.NSCLC的围手术期辅助治疗。

完全切除的Ⅱ期-Ⅲ期NSCLC，推荐含铂两药方案术后辅助化疗3个-4个周期。辅助化疗始于患者术后体力状况基本恢复正常，一般在术后3周-4周开始。

新辅助化疗：对可切除的Ⅲ期NSCLC可选择含铂两药、2个周期的术前新辅助化疗。应当及时评估疗效，并注意判断不良反应，避免增加手术并发症。手术一般在化疗结束后2周-4周进行。术后辅助治疗应当根据术前分期及新辅助化疗疗效，有效者延续原方案或根据患者耐受性酌情调整，无效者则应当更换方案。

4.SCLC的药物治疗。

局限期小细胞肺癌(Ⅱ期-Ⅲ期)推荐放、化疗为主的综合治疗。化疗方案推荐EP或EC方案。

广泛期小细胞肺癌(Ⅳ期)推荐以化疗为主的综合治疗。化疗方案推荐EP、EC或顺铂加拓扑替康(IP)或加伊立替康(IC)。

二线方案推荐拓扑替康。鼓励患者参加新药临床研究。

5.肺癌化疗的原则。

(1) KPS＜60或ECOG＞2的肺癌患者不宜进行化疗。

(2) 白细胞少于3.0×10^9^/L，中性粒细胞少于1.5×10^9^/L、血小板少于6×10^10^/L、红细胞少于2×10^12^/L、血红蛋白低于8.0 g/dL的肺癌患者原则上不宜化疗。

(3) 肺癌患者肝、肾功能异常，实验室指标超过正常值的2倍，或有严重并发症和感染、发热、出血倾向者不宜化疗。

(4) 在化疗中如出现以下情况应当考虑停药或更换方案：

治疗2周期后病变进展，或在化疗周期的休息期中再度恶化者，应当停止原方案，酌情选用其它方案；化疗不良反应达3级-4级，对患者生命有明显威胁时，应当停药，下次治疗时改用其它方案；出现严重的并发症，应当停药，下次治疗时改用其它方案。

(5) 必须强调治疗方案的规范化和个体化。必须掌握化疗的基本要求。除常规应用止吐药物外，铂类药物除卡铂外需要水化和利尿。化疗后每周两次检测血常规。

(6) 化疗的疗效评价参照WHO实体瘤疗效评价标准或RECIST疗效评价标准。

## 五 非小细胞肺癌的分期治疗模式

1.Ⅰ期非小细胞肺癌的综合治疗。

(1) 首选手术治疗，包括肺叶切除加肺门、纵隔淋巴结清除术，可采用开胸或VATS等术式。

(2) 对于肺功能差的患者可以考虑行解剖性肺段或楔形切除术加肺门、纵隔淋巴结清除术。

(3) 完全切除的ⅠA期肺癌患者不适宜行术后辅助化疗。

(4) 完全切除的ⅠB期患者，不推荐常规应用术后辅助化疗。

(5) 切缘阳性的Ⅰ期肺癌推荐再次手术。其它任何原因无法再次手术的患者，推荐术后化疗加放疗。

2.Ⅱ期非小细胞肺癌的综合治疗。

(1) 首选手术治疗，包括肺叶、双肺叶或全肺切除加肺门、纵隔淋巴结清除术。

(2) 对肺功能差的患者可以考虑行解剖性肺段或楔形切除术加肺门、纵隔淋巴结清除术。

(3) 完全性切除的Ⅱ期非小细胞肺癌推荐术后辅助化疗。

(4) 当肿瘤侵犯壁层胸膜或胸壁时应当行整块胸壁切除。切除范围至少距病灶最近的肋骨上下缘各2 cm，受侵肋骨切除长度至少应当距肿瘤5 cm。

(5) 切缘阳性的Ⅱ期肺癌推荐再次手术，其它任何原因无法再次手术的患者，推荐术后化疗加放疗。

3.Ⅲ期非小细胞肺癌的综合治疗。

局部晚期非小细胞肺癌是指TNM分期为Ⅲ期的肺癌。采取综合治疗模式是Ⅲ非小细胞肺癌治疗的最佳选择。将局部晚期NSCLC分为可切除和不可切除两大类。其中：

(1) 可切除的局部晚期非小细胞肺癌包括：

① T_3_N_1_的NSCLC患者，首选手术治疗，术后行辅助化疗。

② N_2_期肺癌患者的手术切除是有争议的。影像学检查发现单组纵隔淋巴结肿大、或两组纵隔淋巴结肿大但没有融合估计能完全切除的病例，推荐行术前纵隔镜检查，明确诊断后行术前新辅助化疗，然后行手术治疗。

③ 一些T_4_N_0-1_的患者：a)相同肺叶内的卫星结节：在新的分期中，此类肺癌为T_3_期，首选治疗为手术切除，也可选择术前新辅助化疗，术后辅助化疗。b)其它可切除之T_4_N_0-1_期非小细胞肺癌，可酌情首选新辅助化疗，也可选择手术切除。如为完全性切除，考虑术后辅助化疗。如切缘阳性，术后行放疗和含铂方案化疗。

④ 肺上沟瘤的治疗：部分可手术患者，建议先行同步放化疗，然后再手术+辅助化疗。对于不能手术的肺上沟瘤，行放疗加化疗。

(2) 不可切除的局部晚期非小细胞肺癌包括：

① 影像学检查提示纵隔的团块状阴影，纵隔镜检查阳性的非小细胞肺癌。

② 大部分的T_4_和N_3_的非小细胞肺癌。

③ T_4_N_2-3_的患者。

④ 胸膜转移结节、恶性胸水和恶性心包积液的患者，新分期已经归类为M_1_，不适于手术切除。部分病例可采用胸腔镜胸膜活检或胸膜固定术。

4.Ⅳ期非小细胞肺癌的治疗。

Ⅳ期肺癌在开始治疗前，建议先获取肿瘤组织进行*EGFR*是否突变的检测，根据*EGFR*突变状况制定相应的治疗策略。

Ⅳ期肺癌以全身治疗为主要手段，治疗目的为提高患者生活质量、延长生命。

(1) 孤立性转移Ⅳ期肺癌的治疗。

① 孤立性脑转移而肺部病变又为可切除的非小细胞肺癌，脑部病变可手术切除或采用立体定向放射治疗，胸部原发病变则按分期治疗原则进行。

② 孤立性肾上腺转移而肺部病变又为可切除的非小细胞肺癌，肾上腺病变可考虑手术切除，胸部原发病变则按分期治疗原则进行。

③ 对侧肺或同侧肺其它肺叶的孤立结节，可分别按两个原发瘤各自的分期进行治疗。

(2) Ⅳ期肺癌的全身治疗。

① *EGFR*敏感突变的Ⅳ期非小细胞肺癌，推荐吉非替尼或厄洛替尼一线治疗。

② 对*EGFR*野生型或突变状况未知的Ⅳ期非小细胞肺癌，如果功能状态评分为PS=0-1，应当尽早开始含铂两药的全身化疗。对不适合铂类治疗的患者，可考虑非铂类两药联合化疗。

③ PS=2的晚期非小细胞肺癌患者应接受单药化疗，但没有证据支持对PS > 2的患者使用细胞毒类药化疗。

④ 目前的证据不支持将年龄因素作为选择化疗方案的依据。

⑤ 一线化疗失败的非小细胞肺癌，推荐多西紫杉醇、培美曲赛二线化疗，以及吉非替尼或厄洛替尼厄二线或三线口服治疗。

⑥ 评分为PS > 2分的Ⅳ期非小细胞肺癌，可酌情仅采用最佳支持治疗。

在全身治疗基础上针对具体的局部情况可以选择恰当的局部治疗方法以求改善症状、提高生活质量。

## 六 小细胞肺癌分期治疗模式

1.Ⅰ期SCLC。手术+辅助化疗(EP/EC 4-6周期)。

2.Ⅱ期-Ⅲ期SCLC：放、化疗联合。

(1) 可选择序贯或同步。

(2) 序贯治疗推荐2周期诱导化疗后同步化、放疗。

(3) 经过规范治疗达到疾病控制者，推荐行预防性脑照射(PCI)。

3.Ⅳ期SCLC：化疗为主的综合治疗以期改善生活质量。

一线推荐EP/EC、IP、IC。规范治疗3个月内疾病复发进展患者推荐进入临床试验。3个-6个月内复发者推荐拓扑替康、伊立替康、吉西他滨或紫杉醇治疗。6个月后疾病进展可选择初始治疗方案。

## 六 诊疗流程和随访

### 一 肺癌诊疗流程

肺癌诊断与治疗的一般流程见附件8。

### 二 随访

对于新发肺癌患者应当建立完整病案和相关资料档案，诊治后定期随访和进行相应检查。具体检查方法包括病史、体检、血液学检查、影像学检查、内镜检查等，旨在监测疾病复发或治疗相关不良反应、评估生活质量等。随访频率为治疗后2年内每3个-6个月随访一次，2年-5年内每6个月随访一次，5年后每年随访一次。

## 附件

1.2004年WHO肺癌组织学类型

2.Karnofsky评分(KPS，百分法)

3.WHO实体瘤疗效评价标准

4.RECIST疗效评价标准

5.急性放射性肺损伤RTOG分级标准

6.Zubrod-ECOG-WHO评分(ZPS，5分法)

7.常用的NSCLC一线化疗方案

8.肺癌诊疗流程

**附件1:** 2004年WHO肺癌组织学类型

鳞状细胞癌
鳞状细胞癌，乳头状亚型
鳞状细胞癌，透明细胞亚型
鳞状细胞癌，小细胞亚型
鳞状细胞癌，基底细胞亚型
小细胞癌
复合性小细胞癌
腺癌
腺癌，混合型
腺泡状腺癌
乳头状腺癌
细支气管肺泡癌
细支气管肺泡癌，非黏液性
细支气管肺泡癌。黏液性
细支气管肺泡癌，黏液及非黏液混合性或不能确定
伴黏液产生的实性腺癌
胎儿性腺癌
黏液性(胶样)腺癌
黏液性囊腺癌
印戒细胞癌
透明细胞腺癌
大细胞癌
大细胞神经内分泌癌
复合性大细胞神经内分泌癌
基底细胞样癌
淋巴上皮样癌
透明细胞癌
大细胞癌伴有横纹肌样表型
腺鳞癌
肉瘤样癌
多形性癌
梭形细胞癌
巨细胞癌
癌肉瘤
肺母细胞瘤
类癌
典型类癌
不典型类癌
唾液腺肿瘤
黏液表皮样癌
腺样囊性癌
上皮-肌上皮癌
癌前病变
原位鳞状细胞癌
不典型腺瘤样增生
弥漫性特发性肺神经内分泌细胞增生

**附件2:** Karnofsky评分(KPS，百分法)

100	健康状况正常，无主诉和明显客观症状和体征。
90	能正常活动，有轻微症状和体征。
80	勉强可进行正常活动，有一些症状或体征。
70	生活可自理，但不能维持正常生活或工作。
60	生活能大部分自理，但偶尔需要别人帮助，不能从事正常工作。
50	生活大部分不能自理，经常治疗和护理。
40	生活不能自理，需专科治疗和护理。
30	生活完全失去自理能力，需要住院和积极的支持治疗。
20	病情严重，必须接受支持治疗。
10	垂危，病情急剧恶化，临近死亡。
0	死亡。

**附件3:** WHO实体瘤疗效评价标准

1.完全缓解(CR)：肿瘤完全消失超过1个月。
2.部分缓解(PR)：肿瘤最大直径及最大垂直直径的乘积缩小达50%，其它病变无增大，持
3.病变稳定(SD)：病变两径乘积缩小不超过50%，增大不超过25%，持续超过1个月。
4.病变进展(PD)：病变两径乘积增大超过25%。

**附件4:** RECIST疗效评价标准

目标病灶的评价：
完全缓解(CR)：所有目标病灶消失。
部分缓解(PR)：目标病灶最长径之和与基线状态比较，至少减少30%。
病变进展(PD)：目标病灶最长径之和与治疗开始之后所记录到的最小的目标病灶最长径之和比较，增加20%，或者出现一个或多个新病灶。
病变稳定(SD)：介于部分缓解和疾病进展之间。
非目标病灶的评价：
完全缓解(CR)：所有非目标病灶消失和肿瘤标志物恢复正常。
未完全缓解/稳定(IR/SD)：存在一个或多个非目标病灶和/或肿瘤标志物持续高于正常值。
病变进展(PD)：出现一个或多个新病灶和/或已有的非目标病灶明确进展。
最佳总疗效的评价：
最佳总疗效的评价是指从治疗开始到疾病进展或复发之间所测量到的最小值。通常，患者最好疗效的分类由病灶测量和确认组成。

**附件5:** 急性放射性肺损伤RTOG分级标准

0级：无变化。
1级：轻度干咳或劳累时呼吸困难。
2级：持续咳嗽需麻醉性止咳药/稍活动即呼吸困难, 但休息时无呼吸困难。
3级：重度咳嗽, 对麻醉性止咳药无效, 或休息时呼吸困难/临床或影像有急性放射性肺炎的证据/间断吸氧或可能需类固醇治疗。
4级：严重呼吸功能不全/持续吸氧或辅助通气治疗。
5级：致命性。

**附件6:** Zubrod-ECOG-WHO评分(ZPS，5分法)

0	正常活动。
1	症状轻，生活自理，能从事轻体力活动。
2	能耐受肿瘤的症状，生活自理，但白天卧床时间不超过50%。
3	肿瘤症状严重，白天卧床时间超过50%，但还能起床站立，部分生活自理。
4	病重卧床不起。
5	死亡。

**附件7:** 常用的NSCLC一线化疗方案

化疗方案	剂量(mg/m^2^)	用药时间	时间及周期
NP：			
长春瑞滨	25	d1，d8	
顺铂	80	d1	q21d×4
TP：			
紫杉醇	135-175	d1	
顺铂	75	d1	
或卡铂	AUC=5-6	d1	q21d×4
GP：			
吉西他滨	1250	d1，d8	
顺铂	75	d1	
或卡铂	AUC=5-6	d1	q21d×4
DP：			
多烯紫杉醇	75	d1	
顺铂	75	d1	
或卡铂	AUC=5-6	d1	q21d×4

**附件8:**